# A case-control study to determine the risk factors for disability among the leprosy cases in Andhra Pradesh, India^☆^

**DOI:** 10.1016/j.abd.2020.11.013

**Published:** 2022-01-04

**Authors:** Srinivas Govindarajulu, Thirumugam Muthuvel, Vivek Lal, Subha Manivannan, Karthikeyan Pandiyambakkam Rajendran, Sudha Seshayyan

**Affiliations:** a Department of Epidemiology, The Tamil Nadu Dr. M.G.R. Medical University, Chennai, India; b Independent Researcher, Hyderabad, India; c Sasakawa-India Leprosy Foundation (S-ILF), New Delhi, India; d JIPMER, Pondicherry, India; e The Tamil Nadu Dr. M.G.R. Medical University, Chennai, India

Dear Editor,

India reports 60% of the world’s new leprosy cases every year.^1^ After the elimination of leprosy as a public health problem in 2005, the National Leprosy Eradication Program (NLEP) was integrated into the existing public Healthcare Provider (HCP) for the diagnosis and early treatment of leprosy.^2^ Despite all efforts, the country reports a significant number of new leprosy cases with disability every year. India alone contributes to 37% of the world’s Grade 2 disability (G2D) cases and 65% of South East Asia.^1^ The proportion of G2D cases among new leprosy cases in the year 2017–2018, as reported from Andhra Pradesh, are 4.86% and 4.71%, respectively.^3^ The delayed presentation is a recognized risk factor for disability, and the present study was done to evaluate the risk factors for disability among adult leprosy cases and measure their strength of association.

A case-control study was conducted in Andhra Pradesh, India, between August 2014 and July 2016. Cases and controls were selected from the NLEP treatment registers from three randomly selected districts, namely Guntur, Krishna, and Kurnool. Cases were defined as adult leprosy patients aged 18 years and older at the time and registered for treatment under NLEP with WHO G2D or G1D. Controls were defined as adult leprosy patients aged 18 years and older at the time and registered for treatment under NLEP with WHO Grade 0 disability (G0D).WHO grading system of leprosy Grade 0, Grade 1, and Grade 2 disability was performed by the NLEP team (Leprosy supervisor and District Leprosy Medical Officer) by assessing the patient’s eyes, hands and feet. The patients from the NLEP treatment register were selected as Cases and Controls.^4^ The sample size calculation and the methodology are described elsewhere.^5^ A pretested questionnaire in the local language (Telugu) was used to record socio-demographic data, information on patient delay, and HCP delay from all the participants. All data obtained were anonymized and analyzed using software STATA version 12.0. Percentages calculated for discrete variables and median calculated for continuous variables with inter-quartile ranges. Variables that were found to be significant in bivariate analysis were subjected to multivariate logistic regression with 95% CI, and a p-value of less than 0.05 was considered statistically significant.

There were lesser males (67%) among the cases than the controls (76%), there were more females among cases (33%) than controls (24%). Socio-demographic characteristics and leprosy types are mentioned in Table 1. The median (calculated instead of mean patient delay because of the skewness of the data) length of patient delay, HCP delay, and total delay in months are presented in Table 2. Risk factors for deformity at the time of diagnosis of leprosy and registration among newly diagnosed leprosy persons are presented in Table 3.

**Table 1:** Socio-demographic characteristics and leprosy type among cases and controls.

Variables	Cases (140)	Controls (140)
	n (%)	n (%)
Gender		
Men	94 (67)	106 (76)
Women	46 (33)	34 (24)
Age at diagnosis (years)		
18–30	54 (39)	60 (43)
30–60	81 (58)	73 (52)
More than 60	5 (4)	7 (5)
Education		
Illiterate	63 (45)	69 (49)
Literate	77 (55)	71 (51)
Marital status		
Unmarried	35 (25)	24 (17)
Married	105 (75)	116 (83)
Residence		
Rural	92 (66)	95 (68)
Urban	48 (34)	45 (32)
Occupation		
Unemployed/Housewife/Student	53 (38)	36 (25)
Daily wage labourer/Agriculture	66 (47)	82 (58)
Salary worker	21 (15)	22 (17)
Leprosy type		
PB	14 (10)	54 (39)
MB	126 (90)	86 (61)
First symptom noticed		
Skin lesion	88 (63)	140 (100)

**Table 2:** Median length of patient delay, HCP delay and total delay in months.

	Cases (140)		Controls (140)		p-value
	Months in median	IQR	Months in median	IQR	
Patient delay	18.0	6.2–30.0	8.0	2.9–18.0	<0.01*
HCP delay	3.2	0.1–12.0	1.0	0.0–4.6	<0.01*
Total delay	24.0	14.0–37.0	13.0	4.9–22.8	<0.01*

IQR, Inter-Quartile Range.

* Significant.

**Table 3:** Patient delay, HCP delay, and related factors as risk factors for deformity at the time of diagnosis of leprosy and registration among newly diagnosed leprosy patients.

	Cases (140)	Controls (140)	Crude Odds Ratio (95% CI)	Adjusted Odds Ratio (95% CI)
	n (%)	n (%)		
**Leprosy type**				
MB	126 (90%)	86 (61%)	5.7 (3.0–10.8)	4.0 (1.9–8.4)
PB	14 (10%)	54 (39%)	1.0	1.0
**Patient delay**				
>3 months	132 (94%)	111 (79%)	5.3 (2.3–11.9)	4.4 (1.6–11.9)
≤3 months	8 (6%)	29 (21%)	1.0	1.0
**HCP delay**				
>1 month	91 (65%)	65 (46%)	2.5 (1.6–4.0)	2.0 (0.94–4.1)
≤1 month	49 (35%)	75 (54%)	1.0	1.0
**First consulted practitioner/public health**				
Non-qualified	38 (27%)	26 (19%)	1.0 (0.5–2.0)	0.4 (0.1–1.2)
Private Practitioner	53 (38%)	48 (34%)	1.9 (1.2–4.6)	1.2 (1.1–2.9)
Public HCP	49 (35%)	66 (47%)	1.0	1.0
**Failure to suspect leprosy by private practitioners in more than 2 visits**				
Yes	50 (35%)	37 (26%)	2.9 (1.7–5.3)	2.2 (1.01–4.9)
No	90 (65%)	103 (74%)	1.0	1.0

Private Practitioner included AYUSH and Allopathy (AYUSH, Ayurveda, Yoga, Unani, Siddha, and Homeopathy); MB, Multi-Bacillary; PB, Pauci-Bacillary; HCP, Health Care Provider.

Detailed analysis of various interplaying factors showed that they could be categorized into either patient-related or HCP-related factors. In this study, the median patient delay was 18 months in cases and eight months in controls. A study in a tertiary hospital in Andhra Pradesh reported 50.5% of leprosy patients had a delay of more than six months before visiting the hospital.^6^ A study in Chhattisgarh reported that 32.5% of G2D/G1D patients had more than 12 months of delay in diagnosis.^7^ However, the delay reported in this study is higher than those reported in other geographical regions in the country. At the international level, Henry et al., in Brazil, reported 34.4% of leprosy patients had a patient delay of fewer than three months.^8^ A study, which observed cases that were reported between 2007 and 2017, in China, reported a mean and the median patient delay of 30.1 months and six months, respectively.^9^ It is found from this study that the patient delay of more than three months poses a significant risk for developing G2D/G1D among new patients with leprosy in Andhra Pradesh.

Though the patient’s delay is the major contributor to the overall delay, the HCP delay cannot be ignored. The prime reason attributed to the health care delay is the lack of skilled health care workers to diagnose the disease. Since leprosy was eliminated as a public health problem, in most countries, less importance is given in the medical curriculum to trainning health care personnel for suspecting leprosy, and often leprosy is misdiagnosed. In India, the health care personnel engaged in the public health care system were better diagnosticians of leprosy compared with those engaged in private practice. This is quite evident from the current study. This study reported that 46% of cases first visited a qualified private health care provider, and 52% of controls first sought care from the public HCP. Various factors like socioeconomic status, education level, accessibility, etc., play a role in health-seeking behavior and the choice of seeking a health care provider. This study reported that when the respondent visited any qualified private health provider more than twice before the diagnosis, the odds of G2D/G1D was two times higher than for those who had visited twice or once. This implies that a significant amount of delay happens with multiple visits to the private sector, and this can serve as a proxy indicator for the missed opportunity for the diagnosis by the private sector.

The main limitation of the current study was its retrospective nature. There might be a “recall bias” introduced to some extent in this study which is inevitable in a case-control study.

In conclusion, patient delay is a vital factor for reducing disability among adult leprosy cases. The high patient delay indicates that the community is not aware of the symptoms and consequences of leprosy, and it also reflects the effectiveness of the educational information and communication strategies and early case detection mechanisms in the state health care services. Also, engaging the private sector for an effective referral system in addition to reducing the number of patients will represent an effective strategy in the early detection and prevention of disability. Community volunteers may be helpful to bridge this gap and link the private sector with the public health system.

## Financial support

This research is part of the Indian Council of Medical Research (ICMR) funded study. We are grateful to ICMR for funding this study.

## Authors’ contributions

Srinivas Govindarajulu: Approval of the final version of the manuscript; conception and planning of the study; statistical analysis; elaboration and writing of the manuscript; obtaining, analyzing and interpreting the data; effective participation in research orientation; critical review of the literature; critical review of the manuscript.

Thirumugam Muthuvel: Approval of the final version of the manuscript; planning of the study, data collection, obtaining, analyzing, statistical analysis and interpreting the data; effective participation in research orientation; critical review of the literature; critical review of the manuscript.

Vivek Lal: Approval of the final version of the manuscript; planning of the study, data collection, obtaining, analyzing, statistical analysis and interpreting the data; effective participation in research orientation; critical review of the literature; critical review of the manuscript.

Subha Manivannan: Approval of the final version of the manuscript; statistical analysis and interpreting the data; critical review of the literature; critical review of the manuscript.

Karthikeyan Pandiyambakkam Rajendran: Approval of the final version of the manuscript; statistical analysis and interpreting the data; critical review of the literature; critical review of the manuscript.

Sudha Seshayyan: Approval of the final version of the manuscript; statistical analysis and interpreting the data; critical review of the literature; critical review of the manuscript.

## Conflicts of interest

None declared.
